# Supplementary material for: Exploring Avenues beyond Revised DSD Functionals: II. Random-Phase Approximation and Scaled MP3 Corrections
Source: J Phys Chem A. 2021 May 21;125(21):4628–38. doi: 10.1021/acs.jpca.1c01295 (PMC8279643; doi:10.1021/acs.jpca.1c01295)
Supplement: Supplementary file 1 — jp1c01295_si_001.pdf [file jp1c01295_si_001.pdf]

Electronic Supporting Information (ESI)

Exploring Avenues Beyond Revised DSD Functionals: II. Random-Phase Approximation and scaled MP3 corrections

Golokesh Santra, [†] *Emmanouil Semidalas,* [†] *and Jan M.L. Martin*[*,†]

[†]Department of Organic Chemistry, Weizmann Institute of Science, 7610001 Reḥovot, Israel.

Email: gershom@weizmann.ac.il

Abbreviations and descriptions used for the GMTKN55 database:

The abbreviations and a concise description of all fifty-five subsets of the GMTKN55 database proposed by Goerigk, Grimme and coworkers[1] are listed in Table S1 below. For a more detailed description of all of them and individual reactions refer to refs. [1–3].

Table S1: Abbreviations used and their descriptions for the 55 datasets in GMTKN55.

Abbreviation	Description
**ACONF**[4]	Relative energies of alkane conformers
**ADIM6**[5]	Interaction energies of n-alkane dimers
**AHB21**[6]	Interaction energies in anion–neutral dimers
**AL2X6**[1]	Dimerisation energies of AlX_3 compounds
**ALK8**[1]	Dissociation and other reactions of alkaline compounds
**ALKBDE10**[7]	Dissociation energies in group-1 and -2 diatomics
**AMINO20X4**[8]	Relative energies in amino acid conformers
**BH76RC**[9]	30 reaction energies of the BH76[10–12] set
**BH76**[10–12]	Barrier heights of hydrogen transfer, heavy atom transfer, nucleophilic substitution, unimolecular and association reactions
**BHDIV10**[1]	Diverse reaction barrier heights
**BHPERI**[1,13,14,15]	Barrier heights of pericyclic reactions
**BHROT27**[1]	Barrier heights for rotation around single bonds
**BSR36**[16,17]	Bond-separation reactions of saturated hydrocarbons
**BUT14DIOL**[18]	Relative energies in butane-1,4-diol conformers
**C60ISO**[19]	Relative energies between C_{60} isomers
**CARBHB12**[1]	Hydrogen-bonded complexes between carbene analogues and H_2O, NH_3, or HCl
**CDIE20**[20]	Double-bond isomerisation energies in cyclic systems
**CHB6**[6]	Interaction energies in cation–neutral dimers
**DARC**[9,21]	Reaction energies of Diels–Alder reactions
**DC13**[22,9,23,24–32]	13 difficult cases for DFT methods
**DIPCS10**[1]	Double-ionisation potentials of closed-shell systems
**FH51**[33,34]	Reaction energies in various (in-)organic systems
**G21EA**[9,35]	Adiabatic electron affinities
**G21IP**[9,35]	Adiabatic ionisation potentials
**G2RC**[7,36]	Reaction energies of selected G2/97 systems
**HAL59**[37,38]	Binding energies in halogenated dimers (incl. halogen bonds)
**HEAVY28**[11]	Noncovalent interaction energies between heavy element hydrides
**HEAVYSB11**[1]	Dissociation energies in heavy-element compounds
**ICONF**[1]	Relative energies in conformers of inorganic systems
**IDISP**[9,39–42]	Intramolecular dispersion interactions
**IL16**[6]	Interaction energies in anion–cation dimers
**INV24**[43]	Inversion/racemisation barrier heights
**ISO34**[39]	Isomerisation energies of small and medium-sized organic molecules
**ISOL24**[44]	Isomerisation energies of large organic molecules
**MB16-43**[1]	Decomposition energies of artificial molecules
**MCONF**[45]	Relative energies in melatonin conformers

**NBPRC**[9,41,46]	Oligomerisations and H_2 fragmentations of NH3/BH3 systems; H_2 activation reactions with PH_3/BH_3 systems
**PA26**[1]	Adiabatic proton affinities (incl. of amino acids)
**PArel**[1]	Relative energies in protonated isomers
**PCONF21**	Relative energies in tri- and tetrapeptide conformers
**PNICO23**[47]	Interaction energies in pnicogen-containing dimers
**PX13**[48]	Proton-exchange barriers in H_2O, NH_3, and HF clusters
**RC21**[1]	Fragmentations and rearrangements in radical cations
**RG18**[1]	Interaction energies in rare-gas complexes
**RSE43**[49]	Radical-stabilisation energies
**S22**[50]	Binding energies of noncovalently bound dimers
**S66**[51]	Binding energies of noncovalently bound dimers
**SCONF**[9,52]	Relative energies of sugar conformers
**SIE4X4**[53]	Self-interaction-error related problems
**TAUT15**[1]	Relative energies in tautomers
**UPU23**[54]	Relative energies between RNA-backbone conformers
**W4-11**[55]	Total atomisation energies
**WATER27**[56]	Binding energies in $(H_2O)_n$, $H+(H_2O)_n$ and $OH-(H_2O)_n$
**WCPT18**[57]	Proton-transfer barriers in uncatalysed and water-catalysed reactions
**YBDE18**[58]	Bond-dissociation energies in ylides

Table S2: WTMAD2 (kcal/mol) and its breakdown into five major subcategories for original and refitted SCS-DRPA75, DSD-PBEdRPA75, DSD-PBEP86dRPA75 and corresponding revDSD functionals with D3BJ and D4 dispersion correction.

Functionals	WTMAD2 (kcal/mol)	THERMO	BARRIERS	LARGE	CONF	INTERMOL
dRPA75	5.072	1.221	0.295	0.931	1.074	1.552
SCS-dRPA75	4.791	0.939	0.310	0.917	1.074	1.552
optSCS-dRPA75	4.712	0.867	0.300	0.919	1.074	1.552
SCS-dRPA75-D3BJ	2.894	0.863	0.299	0.610	0.550	0.572
SCS-dRPA75-D4	2.826	0.848	0.310	0.556	0.533	0.579
optSCS-dRPA75-D3BJ	2.758	0.739	0.287	0.608	0.553	0.571
optSCS-dRPA75-D4	2.700	0.724	0.312	0.534	0.523	0.608
DSD-PBEdRPA$_{75}$-D3BJ	2.377	0.614	0.228	0.513	0.432	0.590
DSD-PBEdRPA$_{75}$-D4	2.321	0.596	0.236	0.477	0.434	0.577
DSD-PBEP86dRPA$_{75}$-D3BJ	2.359	0.613	0.216	0.517	0.433	0.579
DSD-PBEP86dRPA$_{75}$-D4	2.349	0.597	0.226	0.466	0.457	0.603
revDSD-PBE-D3BJ	2.668	0.640	0.299	0.532	0.610	0.586
revDSD-PBE-D4	2.393	0.643	0.290	0.555	0.413	0.491
revDSD-PBEP86-D3BJ	2.367	0.521	0.268	0.552	0.457	0.569
revDSD-PBEP86-D4	2.248	0.545	0.260	0.573	0.406	0.463
ωB97M(2)	2.131	0.430	0.214	0.418	0.577	0.492

Table S3: WTMAD2(kcal/mol), optimized parameters and division of total WTMAD2 into five top-level subsets for DSD-PBEdRPAn and DSD-PBEP86dRPAn with both D3BJ and D4 dispersion correction.

Functional	Dispersion	WTMAD2 (kcal/mol)	Parameters										Five top-level subsets				
			$c_{X,HF}$	$c_{X,DFT}$	$c_{C,DFT}$	co-s	cs-s	s6	s8	cATM	a1	a2	THERMO	BARRIERS	LARGE	CONF	INTERMOL
	D3BJ	8.81	0.00	1.00	0.5877	0.2078	-0.6099	1.4768	0	N/A	0	4.505	2.712	1.214	1.729	1.423	1.733
	D4	7.22	0.00	1.00	0.7861	0.0996	-0.6646	1.5924	0	-0.3616	0.213	4.390	2.643	0.917	1.507	0.969	1.187
	D3BJ	6.09	0.25	0.75	0.5128	0.1971	0.0011	1.0310	0	N/A	0	4.505	1.679	1.038	1.199	0.979	1.195
	D4	4.98	0.25	0.75	0.6942	-0.0781	-0.0098	1.4014	0	0.3157	0.206	4.731	1.776	0.621	1.045	0.725	0.812
	D3BJ	3.37	0.50	0.50	0.3332	0.9030	-0.2004	0.8127	0	N/A	0	4.505	0.927	0.371	0.724	0.634	0.718
	D4	2.85	0.50	0.50	0.4372	0.8435	-0.2262	1.0004	0	0.4909	0.114	5.061	0.775	0.315	0.633	0.518	0.607
**DSD-PBEdRPAn**	D3BJ	2.41	0.68	0.32	0.1901	1.1052	0.3138	0.4563	0	N/A	0	4.505	0.675	0.240	0.517	0.428	0.547
	D4	2.34	0.68	0.32	0.2293	1.0797	0.3264	0.6050	0	0.7034	0.028	5.633	0.632	0.231	0.494	0.443	0.540
	D3BJ	2.38	0.75	0.25	0.1151	1.2072	0.5250	0.3223	0	N/A	0	4.505	0.614	0.228	0.513	0.432	0.590
	D3BJ (a2 opt)	2.33	0.75	0.25	0.1273	1.1991	0.5368	0.3383	0	N/A	0	4.904	0.608	0.228	0.507	0.456	0.531
	D4	2.32	0.75	0.25	0.1339	1.1967	0.5371	0.4257	0	0.6342	-0.145	6.398	0.596	0.236	0.477	0.434	0.577
	D3BJ	3.75	0.90	0.10	-0.0524	1.4564	0.9315	0.0694	0	N/A	0	4.505	0.670	1.148	0.634	0.597	0.702
	D4	3.57	0.90	0.10	-0.0790	1.4760	0.9732	0.1396	0	0.6544	-0.187	6.188	0.666	1.123	0.604	0.486	0.687
	D3BJ	8.55	0.00	1.00	0.5793	0.1823	-0.7157	1.4229	0	N/A	0	4.505	2.322	1.077	1.837	1.457	1.856
	D4	7.07	0.00	1.00	0.7332	0.0865	-0.7483	1.2576	0	-0.8011	0.132	4.427	2.169	0.886	1.706	1.053	1.259
	D3BJ	3.48	0.50	0.50	0.3326	0.8751	-0.2262	0.7488	0	N/A	0	4.505	0.908	0.379	0.806	0.615	0.768
	D4	3.08	0.50	0.50	0.3990	0.8241	-0.2193	0.7759	0	-0.0008	-0.007	5.333	0.810	0.344	0.715	0.549	0.657
	D3BJ	2.43	0.69	0.31	0.1826	1.0984	0.3239	0.4036	0	N/A	0	4.505	0.658	0.235	0.538	0.410	0.590
	D4	2.40	0.69	0.31	0.2023	1.0799	0.3444	0.4709	0	0.3168	-0.144	6.194	0.630	0.230	0.492	0.460	0.590
**DSD-PBEP86dRPAn**	D3BJ	2.36	0.73	0.27	0.1400	1.1525	0.4591	0.3321	0	N/A	0	4.505	0.625	0.219	0.519	0.417	0.578
	D4	2.35	0.73	0.27	0.1482	1.1490	0.4785	0.4043	0	0.3983	-0.234	6.677	0.606	0.221	0.470	0.458	0.596
	D3BJ	2.36	0.75	0.25	0.1092	1.1936	0.5268	0.3012	0	N/A	0	4.505	0.613	0.216	0.517	0.433	0.579
	D3BJ (a2 opt)	2.35	0.75	0.25	0.1204	1.1978	0.5046	0.3156	0	N/A	0	4.752	0.611	0.222	0.512	0.439	0.562
	D4	2.35	0.75	0.25	0.1219	1.1890	0.5281	0.3818	0	0.4571	-0.251	6.772	0.597	0.226	0.466	0.457	0.603
	D3BJ	3.01	0.90	0.10	-0.0553	1.4644	0.9419	0.0744	0	N/A	0	4.505	0.641	0.430	0.621	0.594	0.727
	D4	2.85	0.90	0.10	-0.0744	1.4857	0.9565	0.1606	0	0.5931	-0.258	6.465	0.627	0.427	0.584	0.493	0.715

Table S4: MAD, MSD and RMSD as well as breakdown of total WTMAD2 by each subset for optSCS-dRPA75

subs.name	MAD	MSD	RSMD	dWTMAD2	5MAD/4RMSD
ACONF	0.231	0.231	0.261	0.0717	1.1055
ADIM6	1.153	-1.153	1.256	0.0782	1.1472
AHB21	0.406	0.317	0.447	0.0144	1.1359
AL2X6	3.685	-3.685	3.807	0.0234	1.2098
ALK8	3.571	-2.907	4.728	0.0173	0.9443
ALKBDE10	3.42	-2.721	4.442	0.0129	0.9624
AMINO20X4	0.229	-0.126	0.313	0.2854	0.9151
BH76RC	1.023	0.297	1.324	0.0545	0.9663
BH76	1.095	0.36	2.085	0.1697	0.6563
BHDIV10	0.924	-0.012	1.12	0.0077	1.0319
BHPERI	1.081	0.795	1.245	0.0511	1.0854
BHROT27	0.141	-0.124	0.202	0.023	0.8707
BSR36	2.661	-2.661	2.914	0.2245	1.1413
BUT14DIOL	0.16	-0.12	0.174	0.1393	1.1519
C60ISO	6.907	-6.907	8.798	0.024	0.9813
CARBHB12	0.382	-0.382	0.488	0.0289	0.9801
CDIE20	0.369	0.345	0.446	0.0691	1.0349
CHB6	0.807	-0.807	1.239	0.0069	0.814
DARC	4.363	4.363	4.421	0.0714	1.2336
DC13	5.261	2.576	6.234	0.0472	1.0549
DIPCS10	2.493	2.113	3.085	0.0014	1.0102
FH51	1.766	1.419	2.214	0.1103	0.9971
G21EA	2.036	0.009	2.488	0.0575	1.0228
G21IP	2.494	1.909	3.375	0.0132	0.9235
G2RC	2.019	1.356	2.602	0.0374	0.9698
HAL59	0.567	-0.567	0.646	0.2766	1.0972
HEAVY28	0.355	-0.349	0.392	0.3038	1.1312
HEAVYSB11	2.241	-2.241	2.442	0.0161	1.1468
ICONF	0.265	-0.12	0.355	0.0525	0.9344
IDISP	3.128	1.379	3.797	0.0501	1.0298
IL16	0.652	0.652	0.724	0.0036	1.1259
INV24	0.797	-0.478	1.05	0.0228	0.9487
ISO34	0.909	-0.579	1.194	0.0805	0.9511
ISOL24	2.405	-1.173	3.184	0.1145	0.9441
MB16-43	22.206	-22.206	26.379	0.0774	1.0522
MCONF	0.397	-0.395	0.435	0.1547	1.1417
NBPRC	2.013	1.713	2.555	0.0186	0.9849
PA26	3.974	3.974	4.229	0.0208	1.1746
PAREL	0.543	-0.117	0.919	0.0891	0.7389
PCONF21	0.522	-0.17	0.605	0.2199	1.0786
PNICO23	0.889	-0.889	0.994	0.1817	1.1186
PX13	1.15	-0.86	1.275	0.017	1.1279
RC21	3.976	-3.894	7.192	0.0888	0.691
RG18	0.156	-0.055	0.216	0.1844	0.9075
RSE43	0.786	0.69	1.558	0.1689	0.6308
S22	0.856	-0.856	1.036	0.0979	1.032
S66	0.739	-0.739	0.839	0.3389	1.1011
SCONF	0.249	-0.219	0.287	0.035	1.0845
SIE4X4	11.102	11.102	13.167	0.2	1.0539
TAUT15	0.335	-0.076	0.408	0.0626	1.0264
UPU23	0.427	0.282	0.543	0.0651	0.9817
W4-11	3.024	0.727	4.157	0.0524	0.9091
WATER27	2.905	-2.838	4.531	0.0367	0.8012
WCPT18	0.45	0.118	0.573	0.0088	0.9818
YBDE18	2.362	-1.401	2.831	0.0328	1.0428
**GMTKN55**				**4.7123**	

Table S5: MAD, MSD and RMSD as well as breakdown of total WTMAD2 by each subset for SCS-dRPA74-D3BJ

subs.name	MAD	MSD	RSMD	dWTMAD2	5MAD/4RMSD
ACONF	0.019	0.011	0.03	0.0058	0.7915
ADIM6	0.056	-0.056	0.065	0.0038	1.0862
AHB21	0.187	-0.047	0.282	0.0066	0.8294
AL2X6	0.86	-0.853	0.961	0.0055	1.1187
ALK8	2.455	0.053	3.065	0.0119	1.0011
ALKBDE10	2.648	0.059	3.229	0.01	1.0253
AMINO20X4	0.141	-0.077	0.19	0.1762	0.929
BH76RC	1.06	0.216	1.353	0.0564	0.9789
BH76	1.219	0.048	2.219	0.189	0.6867
BHDIV10	0.952	-0.661	1.14	0.008	1.0439
BHPERI	0.731	-0.731	0.858	0.0345	1.0644
BHROT27	0.107	-0.082	0.166	0.0175	0.8037
BSR36	0.873	-0.873	0.91	0.0736	1.1991
BUT14DIOL	0.038	0.014	0.051	0.033	0.9303
C60ISO	6.31	-6.289	8.256	0.0219	0.9554
CARBHB12	0.114	-0.02	0.177	0.0086	0.8057
CDIE20	0.28	0.25	0.359	0.0524	0.975
CHB6	1.204	-1.204	1.559	0.0102	0.9649
DARC	1.742	1.742	1.834	0.0285	1.1874
DC13	3.316	0.704	4.187	0.0298	0.99
DIPCS10	2.512	2.143	3.105	0.0015	1.0113
FH51	1.262	0.939	1.677	0.0788	0.9406
G21EA	2.586	0.389	3.17	0.073	1.0198
G21IP	3.012	2.272	3.976	0.016	0.9469
G2RC	1.901	1.115	2.55	0.0352	0.9317
HAL59	0.193	-0.042	0.251	0.094	0.9613
HEAVY28	0.136	-0.054	0.157	0.1161	1.0773
HEAVYSB11	1.421	1.421	1.652	0.0102	1.0756
ICONF	0.141	-0.077	0.194	0.0279	0.9075
IDISP	1.058	1.058	1.482	0.017	0.8926
IL16	0.373	-0.284	0.427	0.0021	1.0908
INV24	0.575	-0.071	0.91	0.0164	0.7899
ISO34	0.731	-0.44	0.953	0.0648	0.9596
ISOL24	1.631	-0.589	2.215	0.0776	0.9203
MB16-43	7.26	-3.943	13.123	0.0253	0.6915
MCONF	0.18	0.111	0.201	0.0702	1.1221
NBPRC	1.065	0.619	1.247	0.0098	1.0674
PA26	4.27	4.27	4.492	0.0223	1.1882
PAREL	0.553	-0.115	0.867	0.0907	0.7974
PCONF21	0.276	-0.139	0.346	0.1162	0.9951
PNICO23	0.314	-0.312	0.354	0.0642	1.1096
PX13	1.37	-1.323	1.542	0.0203	1.1105
RC21	3.204	-2.854	6.228	0.0716	0.643
RG18	0.155	0.079	0.198	0.1831	0.982
RSE43	0.816	0.724	1.687	0.1752	0.6046
S22	0.086	0.03	0.112	0.0098	0.9552
S66	0.087	0.024	0.12	0.04	0.9096
SCONF	0.143	-0.067	0.244	0.02	0.7321
SIE4X4	9.667	9.667	11.352	0.1741	1.0645
TAUT15	0.338	-0.009	0.425	0.0631	0.9944
UPU23	0.549	-0.04	0.74	0.0837	0.9261
W4-11	8.958	8.644	10.664	0.1551	1.05
WATER27	2.646	-2.193	6.176	0.0334	0.5356
WCPT18	0.678	-0.431	0.877	0.0132	0.9654
YBDE18	2.789	1.739	3.328	0.0387	1.0474
**GMTKN55**				**2.8942**	

Table S6: MAD, MSD and RMSD as well as breakdown of total WTMAD2 by each subset for optSCS-dRPA75-D3BJ

subs.name	MAD	MSD	RSMD	dWTMAD2	5MAD/4RMSD
ACONF	0.019	0.009	0.029	0.0058	0.8121
ADIM6	0.051	-0.049	0.058	0.0035	1.0936
AHB21	0.187	-0.049	0.282	0.0066	0.8285
AL2X6	0.845	-0.833	0.943	0.0054	1.1197
ALK8	2.461	0.073	3.071	0.0119	1.0017
ALKBDE10	3.326	-2.409	4.085	0.0125	1.0177
AMINO20X4	0.141	-0.077	0.19	0.1759	0.9295
BH76RC	0.944	0.38	1.271	0.0502	0.9283
BH76	1.14	-0.009	2.024	0.1767	0.704
BHDIV10	0.955	-0.666	1.143	0.008	1.0447
BHPERI	0.741	-0.741	0.867	0.0351	1.0685
BHROT27	0.107	-0.082	0.166	0.0174	0.8039
BSR36	0.86	-0.86	0.896	0.0726	1.1999
BUT14DIOL	0.038	0.015	0.051	0.033	0.9322
C60ISO	6.306	-6.285	8.252	0.0219	0.9552
CARBHB12	0.114	-0.017	0.176	0.0086	0.8123
CDIE20	0.279	0.25	0.359	0.0523	0.9742
CHB6	1.207	-1.207	1.562	0.0103	0.9656
DARC	1.724	1.724	1.816	0.0282	1.1863
DC13	3.233	1.379	4.101	0.029	0.9853
DIPCS10	2.512	2.143	3.105	0.0015	1.0113
FH51	1.26	0.936	1.674	0.0786	0.9403
G21EA	1.914	-0.135	2.327	0.054	1.0279
G21IP	2.408	1.805	3.269	0.0128	0.9207
G2RC	1.903	1.114	2.552	0.0352	0.9323
HAL59	0.193	-0.039	0.25	0.0941	0.9629
HEAVY28	0.135	-0.052	0.157	0.1154	1.0757
HEAVYSB11	0.924	-0.356	1.014	0.0067	1.1398
ICONF	0.14	-0.076	0.194	0.0277	0.9061
IDISP	1.056	1.056	1.482	0.0169	0.8908
IL16	0.377	-0.29	0.432	0.0021	1.0912
INV24	0.575	-0.068	0.91	0.0165	0.7897
ISO34	0.73	-0.439	0.951	0.0647	0.9594
ISOL24	1.626	-0.585	2.209	0.0774	0.9203
MB16-43	8.673	-7.092	13.815	0.0302	0.7847
MCONF	0.184	0.115	0.204	0.0715	1.1234
NBPRC	1.058	0.611	1.239	0.0098	1.067
PA26	4.272	4.272	4.494	0.0223	1.1883
PAREL	0.553	-0.115	0.867	0.0908	0.7978
PCONF21	0.279	-0.139	0.351	0.1175	0.994
PNICO23	0.31	-0.308	0.35	0.0634	1.1096
PX13	1.373	-1.327	1.545	0.0203	1.1109
RC21	3.345	-2.929	6.416	0.0747	0.6516
RG18	0.156	0.08	0.198	0.1833	0.9818
RSE43	0.792	0.726	1.553	0.1701	0.6375
S22	0.088	0.036	0.115	0.0101	0.9621
S66	0.087	0.03	0.121	0.04	0.9049
SCONF	0.143	-0.066	0.245	0.0201	0.7302
SIE4X4	11.669	11.669	13.743	0.2102	1.0614
TAUT15	0.338	-0.008	0.425	0.0632	0.9945
UPU23	0.551	-0.042	0.744	0.0842	0.9267
W4-11	2.39	0.374	3.382	0.0414	0.8836
WATER27	2.652	-2.189	6.19	0.0335	0.5355
WCPT18	0.68	-0.435	0.881	0.0133	0.9653
YBDE18	1.416	0.225	1.838	0.0196	0.9627
**GMTKN55**				**2.7583**	

Table S7: MAD, MSD and RMSD as well as breakdown of total WTMAD2 by each subset for DSD-PBEdRPA75-D3BJ

subs.name	MAD	MSD	RSMD	dWTMAD2	5MAD/4RMSD
ACONF	0.023	0.023	0.03	0.0072	0.9599
ADIM6	0.104	-0.104	0.111	0.0071	1.1809
AHB21	0.343	-0.319	0.456	0.0122	0.942
AL2X6	0.425	0.22	0.528	0.0027	1.0051
ALK8	2.964	1.399	3.847	0.0144	0.963
ALKBDE10	4.275	-4.275	5.179	0.0161	1.0318
AMINO20X4	0.103	-0.035	0.133	0.1277	0.962
BH76RC	0.781	0.097	0.929	0.0416	1.0499
BH76	0.925	0.512	1.899	0.1435	0.6093
BHDIV10	0.551	-0.279	0.656	0.0046	1.0504
BHPERI	0.44	-0.217	0.55	0.0208	0.9984
BHROT27	0.081	0.063	0.11	0.0133	0.9256
BSR36	1.134	-1.134	1.202	0.0957	1.1793
BUT14DIOL	0.034	0.009	0.045	0.0296	0.9533
C60ISO	3.249	-2.261	4.229	0.0113	0.9604
CARBHB12	0.168	0.118	0.21	0.0127	1.0022
CDIE20	0.285	0.252	0.407	0.0535	0.8765
CHB6	1.429	-1.429	1.738	0.0122	1.0274
DARC	0.666	-0.645	0.94	0.0109	0.8859
DC13	1.906	-0.191	3.023	0.0171	0.788
DIPCS10	2.345	1.173	3.225	0.0014	0.9089
FH51	0.922	0.053	1.214	0.0575	0.9486
G21EA	1.971	-1.148	2.434	0.0556	1.0124
G21IP	2.155	1.378	2.973	0.0114	0.9061
G2RC	1.953	-0.235	2.692	0.0362	0.9069
HAL59	0.206	-0.038	0.271	0.1007	0.9509
HEAVY28	0.135	-0.05	0.154	0.1152	1.0929
HEAVYSB11	0.802	-0.042	0.918	0.0058	1.0923
ICONF	0.127	-0.002	0.16	0.0251	0.9964
IDISP	0.604	0.576	0.849	0.0097	0.8889
IL16	0.353	-0.279	0.428	0.002	1.0296
INV24	0.571	0.299	0.842	0.0164	0.8485
ISO34	0.593	-0.291	0.765	0.0525	0.9685
ISOL24	0.814	-0.011	1.221	0.0387	0.8332
MB16-43	7.603	-2.351	13.479	0.0265	0.705
MCONF	0.157	0.109	0.175	0.0611	1.12
NBPRC	0.653	-0.057	0.788	0.006	1.0357
PA26	4.49	4.49	4.706	0.0234	1.1926
PAREL	0.408	0.103	0.682	0.0669	0.748
PCONF21	0.194	-0.086	0.233	0.0819	1.0434
PNICO23	0.168	-0.16	0.207	0.0343	1.0125
PX13	0.864	-0.728	1.057	0.0128	1.0224
RC21	2.333	-1.816	5.093	0.0521	0.5727
RG18	0.139	0.044	0.171	0.1637	1.0129
RSE43	0.73	0.711	1.39	0.1568	0.6568
S22	0.145	0.094	0.221	0.0166	0.8185
S66	0.155	0.065	0.219	0.071	0.8851
SCONF	0.118	-0.059	0.188	0.0166	0.7865
SIE4X4	8.949	8.949	10.644	0.1612	1.0509
TAUT15	0.316	0.062	0.366	0.0591	1.0804
UPU23	0.479	0.089	0.663	0.0732	0.9044
W4-11	2.429	-1.252	3.607	0.0421	0.8418
WATER27	3.331	-2.254	7.304	0.0421	0.57
WCPT18	0.843	0.169	1.01	0.0165	1.043
YBDE18	0.762	0.62	1.019	0.0106	0.9351
**GMTKN55**				**2.3768**	

Table S8: MAD, MSD and RMSD as well as breakdown of total WTMAD2 by each subset for DSD-PBEP86dRPA75-D3BJ

subs.name	MAD	MSD	RSMD	dWTMAD2	5MAD/4RMSD
ACONF	0.035	0.035	0.044	0.0108	0.9934
ADIM6	0.047	-0.047	0.058	0.0032	1.0245
AHB21	0.368	-0.346	0.469	0.0131	0.9813
AL2X6	0.443	-0.157	0.526	0.0028	1.0542
ALK8	2.94	0.822	3.696	0.0143	0.9944
ALKBDE10	4.416	-4.416	5.257	0.0167	1.05
AMINO20X4	0.108	-0.035	0.142	0.1345	0.9492
BH76RC	0.873	0.01	1.02	0.0465	1.0702
BH76	0.885	0.344	1.767	0.1375	0.6264
BHDIV10	0.492	-0.185	0.636	0.0041	0.966
BHPERI	0.374	-0.108	0.455	0.0177	1.0291
BHROT27	0.078	0.049	0.103	0.0128	0.9458
BSR36	1.376	-1.376	1.498	0.1163	1.1477
BUT14DIOL	0.04	0.03	0.052	0.035	0.9679
C60ISO	3.185	-2.221	4.147	0.0111	0.9599
CARBHB12	0.186	0.134	0.228	0.0141	1.0209
CDIE20	0.279	0.243	0.402	0.0524	0.8675
CHB6	1.409	-1.409	1.704	0.012	1.0341
DARC	0.482	-0.304	0.724	0.0079	0.8329
DC13	2.103	-0.058	3.137	0.0189	0.8379
DIPCS10	2.389	1.313	3.334	0.0014	0.8956
FH51	0.923	0.097	1.223	0.0577	0.944
G21EA	1.791	-0.872	2.21	0.0506	1.0131
G21IP	2.141	1.403	2.881	0.0114	0.9289
G2RC	1.852	-0.259	2.529	0.0343	0.9152
HAL59	0.201	-0.012	0.256	0.0984	0.9823
HEAVY28	0.132	-0.037	0.155	0.113	1.0659
HEAVYSB11	0.886	-0.485	1.071	0.0064	1.0338
ICONF	0.143	-0.011	0.169	0.0282	1.053
IDISP	0.655	0.655	0.803	0.0105	1.0191
IL16	0.342	-0.25	0.419	0.0019	1.0207
INV24	0.551	0.277	0.822	0.0158	0.8377
ISO34	0.59	-0.299	0.772	0.0523	0.9547
ISOL24	0.877	-0.1	1.334	0.0418	0.8215
MB16-43	8.339	-4.67	14.703	0.0291	0.709
MCONF	0.13	0.086	0.145	0.0505	1.1168
NBPRC	0.653	-0.014	0.843	0.006	0.9675
PA26	4.251	4.251	4.482	0.0222	1.1857
PAREL	0.419	0.082	0.681	0.0688	0.7695
PCONF21	0.175	-0.102	0.218	0.0739	1.0042
PNICO23	0.179	-0.17	0.223	0.0365	1.0009
PX13	0.79	-0.601	0.961	0.0117	1.0278
RC21	2.287	-1.824	5.173	0.0511	0.5525
RG18	0.145	0.083	0.185	0.1709	0.9808
RSE43	0.638	0.613	1.172	0.1373	0.6809
S22	0.174	0.109	0.252	0.0199	0.8624
S66	0.173	0.101	0.249	0.0793	0.8659
SCONF	0.116	-0.082	0.173	0.0163	0.8414
SIE4X4	8.994	8.994	10.763	0.1622	1.0446
TAUT15	0.292	0.052	0.341	0.0547	1.0708
UPU23	0.481	0.09	0.668	0.0735	0.899
W4-11	2.668	-1.568	3.884	0.0463	0.8586
WATER27	1.221	1.221	1.558	0.0164	0.9791
WCPT18	0.861	0.249	1.03	0.0168	1.0448
YBDE18	0.705	0.248	0.884	0.0098	0.997
**GMTKN55**				**2.3588**	

Table S9: MAD, MSD and RMSD as well as breakdown of total WTMAD2 by each subset for DSD-PBEdRPA75-D4

subs.name	MAD	MSD	RSMD	dWTMAD2	5MAD/4RMSD
ACONF	0.033	0.032	0.044	0.0102	0.9243
ADIM6	0.31	-0.31	0.343	0.021	1.1302
AHB21	0.335	-0.305	0.442	0.0119	0.9484
AL2X6	0.374	-0.263	0.44	0.0024	1.0637
ALK8	1.833	-0.418	2.42	0.0089	0.9464
ALKBDE10	4.281	-4.281	5.288	0.0161	1.0121
AMINO20X4	0.128	-0.057	0.172	0.1588	0.929
BH76RC	0.834	0.02	0.96	0.0444	1.0859
BH76	0.977	0.616	1.94	0.1515	0.6299
BHDIV10	0.409	-0.15	0.561	0.0034	0.9117
BHPERI	0.492	-0.093	0.566	0.0233	1.0873
BHROT27	0.085	0.057	0.109	0.014	0.9811
BSR36	0.548	-0.541	0.579	0.0462	1.1827
BUT14DIOL	0.046	0.044	0.059	0.0402	0.9828
C60ISO	3.661	-3.234	5.005	0.0127	0.9144
CARBHB12	0.136	0.059	0.179	0.0103	0.9504
CDIE20	0.359	0.33	0.461	0.0672	0.9732
CHB6	1.482	-1.482	1.839	0.0126	1.0075
DARC	0.656	-0.627	0.879	0.0107	0.9329
DC13	2.129	0.358	2.92	0.0191	0.9112
DIPCS10	2.821	2.168	3.669	0.0016	0.9611
FH51	0.858	0.094	1.145	0.0536	0.9364
G21EA	1.704	-0.609	2.234	0.0481	0.9536
G21IP	2.389	1.836	3.212	0.0127	0.9297
G2RC	1.852	-0.201	2.493	0.0343	0.9286
HAL59	0.27	0.141	0.345	0.1318	0.9791
HEAVY28	0.146	0	0.191	0.1249	0.9565
HEAVYSB11	1.226	0.16	1.349	0.0088	1.1354
ICONF	0.175	-0.056	0.202	0.0345	1.0805
IDISP	0.745	0.349	0.895	0.0119	1.041
IL16	0.361	-0.346	0.403	0.002	1.1197
INV24	0.567	-0.027	0.781	0.0162	0.9071
ISO34	0.631	-0.355	0.79	0.0559	0.9994
ISOL24	0.76	-0.066	1.102	0.0362	0.8621
MB16-43	7.135	-3.526	13.303	0.0249	0.6704
MCONF	0.067	-0.006	0.095	0.026	0.8753
NBPRC	0.803	0.277	0.851	0.0074	1.1793
PA26	3.919	3.919	4.131	0.0205	1.1857
PAREL	0.429	0.163	0.715	0.0703	0.7501
PCONF21	0.155	-0.092	0.2	0.0651	0.9643
PNICO23	0.121	-0.061	0.145	0.0247	1.0373
PX13	0.888	-0.612	1.049	0.0131	1.0585
RC21	2.42	-2.026	5.273	0.0541	0.5737
RG18	0.113	0.049	0.147	0.1336	0.9647
RSE43	0.711	0.675	1.366	0.1526	0.6503
S22	0.131	0.057	0.195	0.015	0.8438
S66	0.177	-0.009	0.236	0.081	0.9364
SCONF	0.129	-0.117	0.192	0.0181	0.8393
SIE4X4	9.028	9.028	10.79	0.1626	1.0458
TAUT15	0.269	0.056	0.319	0.0502	1.0535
UPU23	0.455	0.163	0.596	0.0694	0.9545
W4-11	2.438	-1.203	3.63	0.0422	0.8395
WATER27	0.667	-0.184	0.941	0.0084	0.8871
WCPT18	0.749	0.319	0.926	0.0146	1.0111
YBDE18	0.681	0.225	0.864	0.0094	0.985
**GMTKN55**				**2.3211**	

Table S10: MAD, MSD and RMSD as well as breakdown of total WTMAD2 by each subset for DSD-PBEP86dRPA75-D4

subs.name	MAD	MSD	RSMD	dWTMAD2	5MAD/4RMSD
ACONF	0.043	0.043	0.056	0.0132	0.9519
ADIM6	0.249	-0.249	0.281	0.0169	1.1069
AHB21	0.345	-0.32	0.441	0.0123	0.9793
AL2X6	0.355	-0.185	0.39	0.0023	1.138
ALK8	2.098	-0.721	2.89	0.0102	0.9075
ALKBDE10	4.246	-4.246	5.165	0.016	1.0276
AMINO20X4	0.134	-0.058	0.182	0.1665	0.9179
BH76RC	0.927	-0.055	1.074	0.0494	1.0789
BH76	0.938	0.449	1.806	0.1457	0.6493
BHDIV10	0.358	-0.066	0.558	0.003	0.8021
BHPERI	0.425	-0.004	0.489	0.0201	1.0853
BHROT27	0.082	0.048	0.105	0.0135	0.983
BSR36	0.701	-0.701	0.725	0.0592	1.2086
BUT14DIOL	0.061	0.059	0.073	0.0526	1.035
C60ISO	3.505	-3.026	4.769	0.0122	0.9188
CARBHB12	0.15	0.071	0.197	0.0114	0.9544
CDIE20	0.344	0.312	0.446	0.0645	0.9648
CHB6	1.546	-1.546	1.897	0.0132	1.0185
DARC	0.462	-0.262	0.656	0.0076	0.8807
DC13	2.102	0.282	2.989	0.0189	0.8792
DIPCS10	2.675	2.016	3.63	0.0016	0.9212
FH51	0.867	0.162	1.151	0.0542	0.9416
G21EA	1.628	-0.442	2.089	0.046	0.9742
G21IP	2.305	1.737	3.067	0.0122	0.9393
G2RC	1.805	-0.127	2.466	0.0335	0.915
HAL59	0.288	0.189	0.373	0.1406	0.9655
HEAVY28	0.153	-0.003	0.206	0.131	0.9271
HEAVYSB11	1.317	0.244	1.447	0.0095	1.1382
ICONF	0.182	-0.046	0.213	0.0361	1.0728
IDISP	0.737	0.422	0.863	0.0118	1.0675
IL16	0.318	-0.3	0.361	0.0018	1.103
INV24	0.572	-0.002	0.794	0.0164	0.9004
ISO34	0.606	-0.346	0.786	0.0537	0.9631
ISOL24	0.828	-0.141	1.21	0.0395	0.8549
MB16-43	6.848	-3.144	13.099	0.0239	0.6535
MCONF	0.058	-0.004	0.086	0.0225	0.8361
NBPRC	0.795	0.3	0.828	0.0073	1.1999
PA26	3.661	3.661	3.89	0.0191	1.1765
PAREL	0.431	0.138	0.708	0.0707	0.7606
PCONF21	0.155	-0.101	0.207	0.0656	0.9402
PNICO23	0.114	-0.041	0.138	0.0233	1.03
PX13	0.794	-0.426	0.927	0.0118	1.0714
RC21	2.4	-2.039	5.339	0.0537	0.562
RG18	0.12	0.085	0.162	0.1422	0.9285
RSE43	0.625	0.582	1.154	0.1345	0.6773
S22	0.147	0.065	0.215	0.0168	0.8538
S66	0.188	0.023	0.246	0.0864	0.9583
SCONF	0.142	-0.138	0.19	0.0199	0.9348
SIE4X4	8.946	8.946	10.766	0.1614	1.0387
TAUT15	0.245	0.05	0.297	0.0459	1.0292
UPU23	0.45	0.135	0.613	0.0688	0.9182
W4-11	2.679	-1.491	3.889	0.0465	0.8612
WATER27	0.521	0.502	0.683	0.007	0.9541
WCPT18	0.784	0.431	0.976	0.0153	1.0044
YBDE18	0.687	0.001	0.832	0.0095	1.0318
**GMTKN55**				**2.3487**	

Table S11: MAD, MSD and RMSD as well as breakdown of total WTMAD2 by each subset for optSCS-dRPA75-D4

subs.name	MAD	MSD	RSMD	dWTMAD2	5MAD/4RMSD
ACONF	0.031	0.03	0.046	0.0098	0.8459
ADIM6	0.134	-0.134	0.153	0.0091	1.0882
AHB21	0.185	-0.106	0.269	0.0065	0.8575
AL2X6	0.883	-0.883	0.942	0.0056	1.1712
ALK8	2.171	-1.253	3.015	0.0105	0.9001
ALKBDE10	3.404	-2.362	4.172	0.0128	1.02
AMINO20X4	0.155	-0.079	0.21	0.1934	0.9242
BH76RC	0.96	0.371	1.297	0.0511	0.9247
BH76	1.182	-0.015	2.044	0.1832	0.7225
BHDIV10	0.942	-0.751	1.151	0.0079	1.0231
BHPERI	1.04	-1.04	1.143	0.0492	1.1376
BHROT27	0.113	-0.065	0.168	0.0184	0.8375
BSR36	0.197	-0.079	0.235	0.0166	1.0492
BUT14DIOL	0.045	0.039	0.057	0.0393	0.9902
C60ISO	6.848	-6.848	8.765	0.0238	0.9767
CARBHB12	0.113	-0.03	0.171	0.0085	0.8286
CDIE20	0.304	0.28	0.365	0.057	1.0432
CHB6	1.153	-1.153	1.637	0.0098	0.8801
DARC	1.229	1.229	1.371	0.0201	1.1213
DC13	2.976	1.499	3.706	0.0267	1.0037
DIPCS10	2.701	2.533	3.356	0.0016	1.0062
FH51	1.218	0.892	1.647	0.076	0.9242
G21EA	1.811	0.149	2.271	0.0511	0.9965
G21IP	2.466	1.916	3.326	0.0131	0.9268
G2RC	1.963	1.189	2.628	0.0363	0.9338
HAL59	0.272	0.178	0.345	0.1326	0.986
HEAVY28	0.177	-0.03	0.216	0.1513	1.0222
HEAVYSB11	0.926	-0.094	1.051	0.0067	1.1021
ICONF	0.182	-0.099	0.232	0.0359	0.9808
IDISP	0.949	0.817	1.459	0.0152	0.8134
IL16	0.612	-0.612	0.654	0.0034	1.1684
INV24	0.697	-0.316	0.981	0.0199	0.888
ISO34	0.729	-0.454	0.959	0.0646	0.9503
ISOL24	1.462	-0.427	2.063	0.0696	0.8859
MB16-43	7.063	-5.348	13.044	0.0246	0.6768
MCONF	0.074	0.017	0.091	0.0288	1.0222
NBPRC	1.087	0.72	1.215	0.01	1.1183
PA26	3.687	3.687	3.898	0.0193	1.1822
PAREL	0.527	-0.063	0.845	0.0865	0.7797
PCONF21	0.263	-0.196	0.371	0.1109	0.8864
PNICO23	0.15	-0.134	0.194	0.0306	0.9646
PX13	1.34	-1.285	1.509	0.0198	1.1104
RC21	3.333	-2.881	6.391	0.0745	0.652
RG18	0.143	0.097	0.197	0.169	0.9081
RSE43	0.796	0.724	1.554	0.171	0.6406
S22	0.122	0.025	0.17	0.014	0.8987
S66	0.149	0.002	0.19	0.0682	0.9772
SCONF	0.121	-0.065	0.189	0.017	0.8034
SIE4X4	11.636	11.636	13.732	0.2096	1.0593
TAUT15	0.317	0.041	0.386	0.0593	1.0272
UPU23	0.474	-0.025	0.653	0.0723	0.9069
W4-11	2.384	0.164	3.353	0.0413	0.8888
WATER27	0.42	0.059	0.514	0.0053	1.0218
WCPT18	0.668	-0.409	0.856	0.0131	0.9756
YBDE18	1.311	0.388	1.793	0.0182	0.9139
**GMTKN55**				**2.7001**	

Table S12: MAD, MSD and RMSD as well as breakdown of total WTMAD2 by each subset for SCS-dRPA75-D4

subs.name	MAD	MSD	RSMD	dWTMAD2	5MAD/4RMSD
ACONF	0.025	0.017	0.034	0.0077	0.8971
ADIM6	0.129	-0.129	0.152	0.0088	1.0592
AHB21	0.171	-0.064	0.263	0.006	0.8122
AL2X6	1.257	-1.257	1.314	0.008	1.196
ALK8	2.358	-1.426	3.096	0.0114	0.952
ALKBDE10	2.756	-0.147	3.383	0.0104	1.0185
AMINO20X4	0.161	-0.087	0.218	0.2008	0.9234
BH76RC	1.079	0.191	1.383	0.0574	0.975
BH76	1.241	0.099	2.234	0.1923	0.6941
BHDIV10	0.897	-0.611	1.084	0.0075	1.0341
BHPERI	0.852	-0.852	0.971	0.0403	1.0975
BHROT27	0.114	-0.08	0.178	0.0187	0.8058
BSR36	0.235	-0.133	0.268	0.0198	1.096
BUT14DIOL	0.052	0.046	0.063	0.045	1.0329
C60ISO	6.953	-6.953	8.87	0.0242	0.9799
CARBHB12	0.11	-0.043	0.176	0.0083	0.7835
CDIE20	0.313	0.286	0.374	0.0585	1.0437
CHB6	1.149	-1.149	1.624	0.0098	0.8844
DARC	1.574	1.574	1.66	0.0258	1.1854
DC13	3.186	1.112	3.896	0.0286	1.0221
DIPCS10	2.646	2.428	3.282	0.0015	1.0076
FH51	1.25	0.955	1.676	0.078	0.9324
G21EA	2.595	0.612	3.169	0.0732	1.0234
G21IP	3.054	2.357	4.029	0.0162	0.9474
G2RC	1.868	1.213	2.565	0.0346	0.9103
HAL59	0.255	0.158	0.322	0.1246	0.9907
HEAVY28	0.161	0.002	0.199	0.1382	1.016
HEAVYSB11	1.316	1.316	1.669	0.0095	0.9862
ICONF	0.19	-0.12	0.242	0.0375	0.9817
IDISP	1.041	0.88	1.481	0.0167	0.8785
IL16	0.516	-0.516	0.565	0.0029	1.1414
INV24	0.721	-0.34	1	0.0206	0.9015
ISO34	0.751	-0.471	0.985	0.0665	0.9525
ISOL24	1.522	-0.545	2.124	0.0725	0.8954
MB16-43	7.236	-4.89	13.578	0.0252	0.6662
MCONF	0.066	-0.006	0.093	0.0256	0.8798
NBPRC	1.207	0.818	1.318	0.0111	1.1442
PA26	3.761	3.761	3.982	0.0196	1.1804
PAREL	0.528	-0.076	0.857	0.0865	0.7698
PCONF21	0.261	-0.19	0.367	0.1101	0.8906
PNICO23	0.183	-0.164	0.244	0.0374	0.9387
PX13	1.26	-1.188	1.439	0.0186	1.0946
RC21	3.284	-2.974	6.349	0.0734	0.6467
RG18	0.142	0.102	0.195	0.1676	0.9098
RSE43	0.825	0.73	1.688	0.1773	0.6112
S22	0.097	0.047	0.149	0.0111	0.8144
S66	0.125	0.016	0.166	0.0573	0.9387
SCONF	0.135	-0.09	0.218	0.0189	0.7743
SIE4X4	9.611	9.611	11.323	0.1731	1.061
TAUT15	0.302	0	0.387	0.0565	0.9768
UPU23	0.465	0.015	0.641	0.0709	0.9066
W4-11	8.565	8.179	10.242	0.1483	1.0454
WATER27	0.535	-0.19	0.699	0.0068	0.9566
WCPT18	0.615	-0.323	0.788	0.012	0.9758
YBDE18	2.657	1.537	3.161	0.0368	1.0507
**GMTKN55**				**2.8262**	

Table S13: MAD, MSD and RMSD as well as breakdown of total WTMAD2 by each subset for ωDSD3-PBEP86-D3BJ

subs.name	Nsys	Nskip	MAD	MSD	RSMD	dWTMAD2	5MAD/4RMSD
ACONF	15	0	0.017	0.007	0.02	0.0055	1.056
ADIM6	6	0	0.02	-0.004	0.024	0.0014	1.0353
AHB21	21	0	0.17	-0.143	0.262	0.0062	0.8119
AL2X6	6	0	0.278	-0.238	0.321	0.0018	1.0827
ALK8	8	0	1.435	0.747	1.877	0.0072	0.9559
ALKBDE10	10	0	3.097	-2.998	4.011	0.0121	0.9652
AMINO20X4	80	0	0.091	-0.038	0.123	0.1176	0.9302
BH76RC	30	0	0.624	0.223	0.84	0.0343	0.9293
BH76	76	0	0.755	-0.128	1.293	0.1209	0.73
BHDIV10	10	0	0.717	-0.44	0.838	0.0062	1.0692
BHPERI	26	0	0.374	0.171	0.456	0.0183	1.0249
BHROT27	27	0	0.074	0.034	0.1	0.0126	0.9284
BSR36	36	0	0.708	-0.708	0.781	0.0617	1.1335
BUT14DIOL	64	0	0.046	0.02	0.057	0.0412	1.0022
CARBHB12	12	0	0.287	0.287	0.353	0.0223	1.0152
CDIE20	20	0	0.168	0.12	0.274	0.0325	0.7675
CHB6	6	0	1.083	-1.083	1.297	0.0095	1.044
DARC	14	0	0.561	0.471	0.646	0.0095	1.0864
DC13	13	0	2.464	-0.031	3.297	0.0229	0.9343
DIPCS10	10	0	4.941	-4.941	5.1	0.003	1.2109
FH51	51	0	0.662	0.128	0.862	0.0427	0.9602
G21EA	25	0	2.707	-2.707	3.013	0.0789	1.1229
G21IP	36	0	2.09	-1.455	2.477	0.0115	1.0543
G2RC	25	0	1.422	0.357	2.146	0.0272	0.8281
HAL59	59	0	0.228	0.039	0.285	0.1147	0.9988
HEAVY28	28	0	0.087	0.022	0.124	0.0769	0.8785
HEAVYSB11	11	0	1.216	-1.216	1.369	0.009	1.1108
ICONF	17	0	0.085	0.02	0.108	0.0173	0.9859
IDISP	5	1	0.349	0.349	0.399	0.0041	1.0939
IL16	16	0	0.333	0.282	0.426	0.0019	0.9772
INV24	21	3	0.483	0.123	1.022	0.013	0.5906
ISO34	34	0	0.304	-0.205	0.446	0.0279	0.8536
ISOL24	14	10	0.739	-0.333	0.995	0.021	0.9276
MB16-43	43	0	7.077	-6.167	8.534	0.0255	1.0366
MCONF	51	0	0.155	0.141	0.181	0.0625	1.0746
NBPRC	6	0	0.372	-0.197	0.437	0.0036	1.0657
PA26	26	0	1.423	1.422	1.688	0.0077	1.0541
PAREL	20	0	0.315	-0.013	0.522	0.0533	0.7532
PCONF21	18	0	0.165	-0.082	0.196	0.0719	1.055
PNICO23	23	0	0.119	0.089	0.168	0.0252	0.8902
PX13	13	0	1.564	-1.564	1.659	0.0239	1.1782
RC21	21	0	0.714	-0.292	0.93	0.0165	0.959
RG18	18	0	0.073	-0.03	0.095	0.089	0.9643
RSE43	43	0	0.289	0.153	0.518	0.0641	0.6969
S22	22	0	0.139	-0.032	0.181	0.0164	0.955
S66	66	0	0.12	0.037	0.156	0.0567	0.9603
SCONF	17	0	0.122	0.014	0.184	0.0176	0.8253
SIE4X4	16	0	5.157	5.157	5.886	0.096	1.0952
TAUT15	15	0	0.417	-0.173	0.5	0.0805	1.042
W4-11	140	0	2.199	-1.803	2.98	0.0394	0.9225
WATER27	23	4	0.742	0.722	0.837	0.0113	1.1085
WCPT18	18	0	0.989	-0.793	1.185	0.02	1.0431
YBDE18	18	0	0.614	-0.354	0.755	0.0088	1.0161
**GMTKN55**	1449	50				**1.7826**	

Table S14: MAD, MSD and RMSD as well as breakdown of total WTMAD2 by each subset for ωDSD3-PBEP86-D4

subs.name	Nsys	Nskip	MAD	MSD	RSMD	dWTMAD2	5MAD/4RMSD
ACONF	15	0	0.018	0.01	0.023	0.0057	0.9568
ADIM6	6	0	0.032	-0.032	0.041	0.0022	0.9754
AHB21	21	0	0.174	-0.163	0.257	0.0064	0.8483
AL2X6	6	0	0.895	-0.895	0.901	0.0059	1.2407
ALK8	8	0	1.599	-0.724	1.881	0.008	1.0622
ALKBDE10	10	0	3.186	-3.115	4.195	0.0124	0.9492
AMINO20X4	80	0	0.098	-0.039	0.129	0.1266	0.9531
BH76RC	30	0	0.618	0.201	0.842	0.034	0.9172
BH76	76	0	0.753	-0.139	1.287	0.1205	0.7312
BHDIV10	10	0	0.696	-0.365	0.833	0.006	1.0451
BHPERI	26	0	0.423	0.319	0.511	0.0207	1.0347
BHROT27	27	0	0.077	0.021	0.097	0.013	0.9956
BSR36	36	0	0.519	-0.519	0.558	0.0452	1.1634
BUT14DIOL	64	0	0.073	0.071	0.082	0.0656	1.1195
CARBHB12	12	0	0.252	0.252	0.32	0.0196	0.982
CDIE20	20	0	0.201	0.157	0.301	0.0388	0.8328
CHB6	6	0	0.767	-0.767	1.015	0.0067	0.945
DARC	14	0	0.708	0.669	0.799	0.012	1.1079
DC13	13	0	2.371	0.328	3.155	0.022	0.9393
DIPCS10	10	0	4.594	-4.594	4.748	0.0028	1.2096
FH51	51	0	0.69	0.178	0.892	0.0445	0.967
G21EA	25	0	2.502	-2.5	2.82	0.073	1.1091
G21IP	36	0	1.98	-1.321	2.379	0.0109	1.0405
G2RC	25	0	1.348	0.364	2.055	0.0258	0.8197
HAL59	59	0	0.228	0.085	0.278	0.115	1.0264
HEAVY28	28	0	0.105	0.03	0.139	0.0927	0.9397
HEAVYSB11	11	0	1.706	-1.706	1.814	0.0127	1.1757
ICONF	17	0	0.101	-0.007	0.134	0.0207	0.9476
IDISP	5	1	0.419	0.41	0.538	0.0049	0.9731
IL16	16	0	0.22	0.153	0.282	0.0013	0.9746
INV24	21	3	0.594	-0.056	1.055	0.0159	0.7043
ISO34	34	0	0.334	-0.245	0.488	0.0306	0.8551
ISOL24	14	10	0.778	-0.473	1.022	0.0221	0.9518
MB16-43	43	0	9.938	-9.55	11.348	0.0358	1.0947
MCONF	51	0	0.088	0.062	0.108	0.0352	1.0114
NBPRC	6	0	0.363	-0.035	0.42	0.0035	1.082
PA26	26	0	1.113	1.107	1.397	0.006	0.996
PAREL	20	0	0.308	0.003	0.531	0.0521	0.7245
PCONF21	18	0	0.116	-0.079	0.15	0.0504	0.9634
PNICO23	23	0	0.079	0.074	0.108	0.0167	0.9132
PX13	13	0	1.527	-1.527	1.634	0.0233	1.1682
RC21	21	0	0.682	-0.372	0.943	0.0157	0.9044
RG18	18	0	0.065	-0.02	0.08	0.0797	1.019
RSE43	43	0	0.282	0.099	0.481	0.0625	0.7324
S22	22	0	0.104	0.025	0.137	0.0123	0.9506
S66	66	0	0.118	0.061	0.159	0.0558	0.9281
SCONF	17	0	0.123	0.008	0.192	0.0178	0.8002
SIE4X4	16	0	5.183	5.183	5.924	0.0965	1.0937
TAUT15	15	0	0.385	-0.145	0.462	0.0743	1.0414
W4-11	140	0	2.225	-1.786	3.066	0.0398	0.9073
WATER27	23	4	0.638	0.612	0.712	0.0097	1.1198
WCPT18	18	0	0.94	-0.754	1.12	0.019	1.0495
YBDE18	18	0	0.874	-0.651	0.96	0.0125	1.1386
**GMTKN55**	1449	50				**1.7629**	

Table S15: MAD, MSD and RMSD as well as breakdown of total WTMAD2 by each subset for DSD3-PBEP86-D3BJ

subs.name	Nsys	Nskip	MAD	MSD	RSMD	dWTMAD2	5MAD/4RMSD
ACONF	15	0	0.021	0.012	0.028	0.0068	0.9569
ADIM6	6	0	0.031	-0.031	0.039	0.0022	0.9954
AHB21	21	0	0.202	-0.101	0.286	0.0074	0.8814
AL2X6	6	0	0.22	-0.216	0.351	0.0014	0.7834
ALK8	8	0	1.365	1.321	1.858	0.0068	0.9182
ALKBDE10	10	0	3.008	-3.008	3.474	0.0117	1.0824
AMINO20X4	80	0	0.107	-0.047	0.137	0.1373	0.9746
BH76RC	30	0	0.731	0.219	0.868	0.0402	1.0524
BH76	76	0	0.928	-0.162	1.582	0.1487	0.7334
BHDIV10	10	0	0.938	-0.702	1.089	0.0081	1.0762
BHPERI	26	0	0.319	-0.196	0.405	0.0156	0.9852
BHROT27	27	0	0.074	0.057	0.093	0.0124	0.9883
BSR36	36	0	1.597	-1.597	1.853	0.1393	1.0777
BUT14DIOL	64	0	0.06	-0.021	0.074	0.0542	1.0184
CARBHB12	12	0	0.397	0.397	0.469	0.031	1.0582
CDIE20	20	0	0.26	0.244	0.324	0.0503	1.0034
CHB6	6	0	1.106	-1.106	1.287	0.0097	1.0736
DARC	14	0	0.962	0.906	1.09	0.0163	1.1029
DC13	13	0	2.783	-0.405	3.724	0.0258	0.9343
DIPCS10	10	0	4.166	-4.166	4.401	0.0025	1.1832
FH51	51	0	0.821	0.224	1.098	0.053	0.9347
G21EA	25	0	2.297	-2.001	2.624	0.067	1.0946
G21IP	36	0	2.013	-0.963	2.458	0.011	1.0238
G2RC	25	0	1.697	0.198	2.355	0.0325	0.9006
HAL59	59	0	0.259	0.147	0.35	0.1305	0.9261
HEAVY28	28	0	0.143	0.139	0.182	0.1264	0.9822
HEAVYSB11	11	0	0.834	-0.256	0.931	0.0062	1.1203
ICONF	17	0	0.078	-0.004	0.099	0.0159	0.9812
IDISP	5	1	0.968	0.888	1.416	0.0114	0.8544
IL16	16	0	0.45	0.45	0.533	0.0026	1.0565
INV24	21	3	0.425	0.038	0.874	0.0114	0.607
ISO34	34	0	0.345	-0.188	0.497	0.0316	0.8689
ISOL24	14	10	1.239	-0.312	1.881	0.0352	0.8233
MB16-43	43	0	7.676	-7.136	9.679	0.0276	0.9913
MCONF	51	0	0.092	0.051	0.117	0.0372	0.9865
NBPRC	6	0	0.404	-0.302	0.545	0.0038	0.926
PA26	26	0	1.586	1.586	1.881	0.0086	1.0542
PAREL	20	0	0.376	-0.004	0.638	0.0636	0.7356
PCONF21	18	0	0.176	-0.061	0.216	0.0766	1.0178
PNICO23	23	0	0.25	0.242	0.333	0.0527	0.9366
PX13	13	0	1.428	-1.428	1.491	0.0218	1.1976
RC21	21	0	0.849	-0.503	1.008	0.0196	1.0521
RG18	18	0	0.068	-0.017	0.085	0.0826	1.0008
RSE43	43	0	0.543	0.468	1.081	0.1205	0.6283
S22	22	0	0.164	-0.015	0.209	0.0194	0.9801
S66	66	0	0.116	0.054	0.153	0.0551	0.9518
SCONF	17	0	0.102	-0.084	0.133	0.0148	0.9628
SIE4X4	16	0	5.329	5.329	6.12	0.0992	1.0883
TAUT15	15	0	0.42	-0.22	0.514	0.081	1.0208
W4-11	140	0	2.406	-1.831	3.427	0.043	0.8774
WATER27	23	4	0.57	0.458	0.697	0.0087	1.0233
WCPT18	18	0	0.878	-0.812	1.15	0.0177	0.955
YBDE18	18	0	0.584	-0.1	0.732	0.0084	0.9977
**GMTKN55**	1449	50				**2.1247**	

Table S16: MAD, MSD and RMSD as well as breakdown of total WTMAD2 by each subset for DSD3-PBEP86-D4

subs.name	Nsys	Nskip	MAD	MSD	RSMD	dWTMAD2	5MAD/4RMSD
ACONF	15	0	0.022	0.013	0.03	0.0071	0.9202
ADIM6	6	0	0.029	-0.013	0.039	0.002	0.9269
AHB21	21	0	0.177	-0.097	0.265	0.0065	0.836
AL2X6	6	0	1.546	-1.546	1.567	0.0101	1.2332
ALK8	8	0	1.465	-0.999	1.75	0.0073	1.0462
ALKBDE10	10	0	3.179	-3.179	3.723	0.0124	1.0675
AMINO20X4	80	0	0.114	-0.051	0.145	0.1463	0.9803
BH76RC	30	0	0.734	0.203	0.879	0.0404	1.044
BH76	76	0	0.899	-0.102	1.587	0.144	0.7083
BHDIV10	10	0	0.778	-0.522	0.993	0.0067	0.9801
BHPERI	26	0	0.23	-0.018	0.274	0.0113	1.0515
BHROT27	27	0	0.065	0.038	0.081	0.0109	1.0014
BSR36	36	0	1.532	-1.532	1.761	0.1336	1.0875
BUT14DIOL	64	0	0.049	0.039	0.066	0.0441	0.9279
CARBHB12	12	0	0.336	0.336	0.407	0.0262	1.0322
CDIE20	20	0	0.294	0.28	0.358	0.0568	1.025
CHB6	6	0	0.654	-0.523	0.794	0.0057	1.0287
DARC	14	0	1.173	1.165	1.303	0.0198	1.1254
DC13	13	0	2.709	0.186	3.299	0.0251	1.0264
DIPCS10	10	0	3.924	-3.924	4.135	0.0024	1.1863
FH51	51	0	0.852	0.278	1.131	0.055	0.942
G21EA	25	0	2.168	-1.828	2.515	0.0632	1.0779
G21IP	36	0	1.968	-0.887	2.427	0.0108	1.0137
G2RC	25	0	1.524	0.239	2.149	0.0291	0.8862
HAL59	59	0	0.192	0.064	0.245	0.0968	0.9821
HEAVY28	28	0	0.102	0.037	0.139	0.09	0.9153
HEAVYSB11	11	0	1.445	-1.445	1.605	0.0107	1.125
ICONF	17	0	0.113	-0.031	0.159	0.0231	0.8874
IDISP	5	1	1.047	0.917	1.409	0.0123	0.9289
IL16	16	0	0.315	0.309	0.373	0.0018	1.0553
INV24	21	3	0.576	-0.139	0.943	0.0154	0.7629
ISO34	34	0	0.378	-0.243	0.556	0.0346	0.8505
ISOL24	14	10	1.307	-0.497	1.871	0.0372	0.8733
MB16-43	43	0	14.657	-14.657	16.319	0.0528	1.1227
MCONF	51	0	0.074	-0.001	0.089	0.0299	1.0379
NBPRC	6	0	0.531	-0.04	0.67	0.0051	0.99
PA26	26	0	1.264	1.259	1.574	0.0068	1.0036
PAREL	20	0	0.37	0.004	0.654	0.0627	0.7067
PCONF21	18	0	0.108	-0.055	0.139	0.0472	0.9748
PNICO23	23	0	0.111	0.106	0.132	0.0233	1.0492
PX13	13	0	1.374	-1.374	1.442	0.021	1.1906
RC21	21	0	0.93	-0.744	1.164	0.0215	0.9984
RG18	18	0	0.068	-0.013	0.081	0.0824	1.0446
RSE43	43	0	0.559	0.478	1.105	0.124	0.6321
S22	22	0	0.122	0.074	0.142	0.0145	1.0787
S66	66	0	0.136	0.1	0.165	0.0643	1.0315
SCONF	17	0	0.096	-0.091	0.137	0.0139	0.8745
SIE4X4	16	0	5.295	5.295	6.114	0.0985	1.0827
TAUT15	15	0	0.407	-0.224	0.509	0.0786	1
W4-11	140	0	2.49	-1.895	3.493	0.0446	0.8912
WATER27	23	4	0.49	0.349	0.584	0.0074	1.0483
WCPT18	18	0	0.809	-0.74	1.054	0.0163	0.9591
YBDE18	18	0	0.9	-0.502	1.038	0.0129	1.0838
**GMTKN55**	1449	50				**2.0266**	

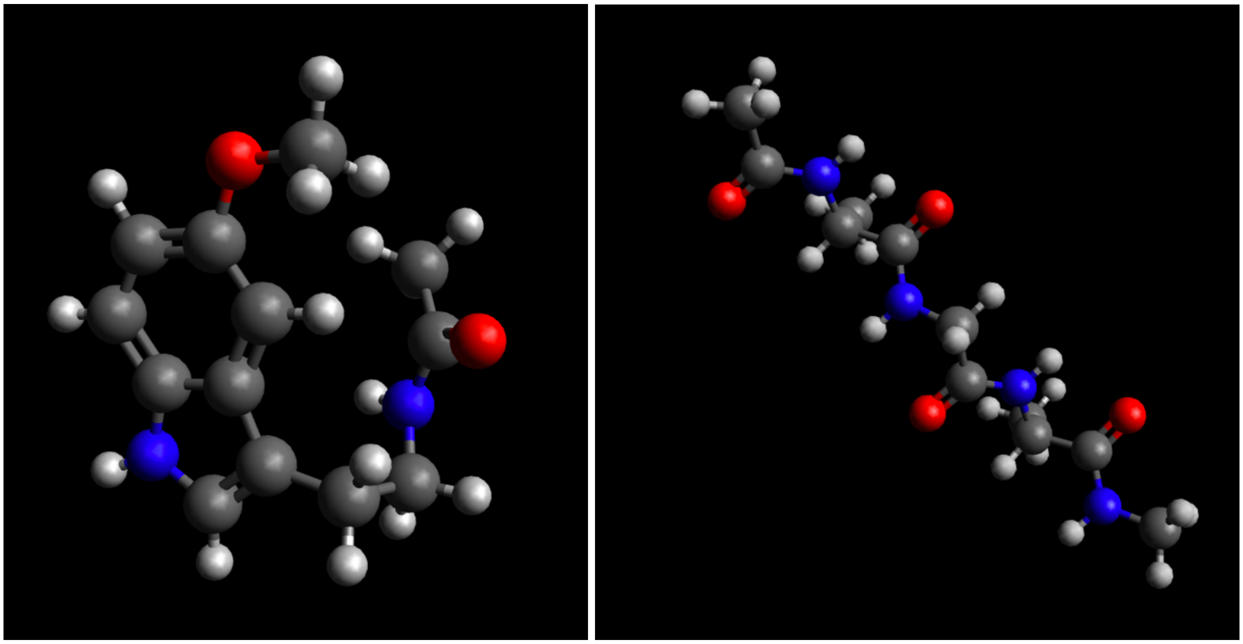

Figure S1. Structures of two systems upon which we experimented the computational time requirements for different functionals. $MCONF_1$ (left), $PCONF21_{GLY(ab)}$ (right).

ORCA sample input files for CO:

1. revDSD-PBEP86-D3(BJ)

```
!PAL8

%MaxCore 2500

! RKS def2-QZVPP def2-qzvpp/c def2/j tightscf rijcosx GRID6 NoPOP d3bj

%method
   Exchange X_PBE
   Correlation C_P86
ScalHFX 0.69
ScalDFX 0.31
ScalGGAC 0.4296
ScalLDAC 0.4296
   ScalMP2C 1.0
   D3S6 0.4377
   D3A2 5.5
   D3S8 0.0
   D3A1 0.0
end

%mp2
DoSCS true
   PS 0.5785
   PT 0.0799
end

*xyz 0 1
   C     0.000000    0.000000    0.000000
   O     0.000000    0.000000    1.131400
*
```

2. revDSD-PBEPBE-D3(BJ)

```
!PAL8

%MaxCore 2500

! RKS def2-QZVPP def2-qzvpp/c def2/j tightscf rijcosx GRID6 NoPOP d3bj
%method
   Exchange X_PBE
   Correlation C_PBE
ScalHFX 0.68
ScalDFX 0.32
ScalGGAC 0.4528
ScalLDAC 0.4528
   ScalMP2C 1.0
   D3S6 0.5746
   D3A2 5.50
   D3S8 0.0
   D3A1 0.0
end
```

```
%mp2
DoSCS true
   PS 0.5845
   PT 0.0711
end

*xyz 0 1
   C     0.000000    0.000000    0.000000
   O     0.000000    0.000000    1.131400
*
```

References

(1) Goerigk, L.; Hansen, A.; Bauer, C.; Ehrlich, S.; Najibi, A.; Grimme, S. A Look at the Density Functional Theory Zoo with the Advanced GMTKN55 Database for General Main Group Thermochemistry, Kinetics and Noncovalent Interactions. *Phys. Chem. Chem. Phys.* **2017**, *19*, 32184–32215.

(2) Goerigk, L.; Grimme, S. A Thorough Benchmark of Density Functional Methods for General Main Group Thermochemistry, Kinetics, and Noncovalent Interactions. *Phys. Chem. Chem. Phys.* **2011**, *13*, 6670–6688.

(3) Goerigk, L.; Mehta, N. A Trip to the Density Functional Theory Zoo: Warnings and Recommendations for the User*. *Aust. J. Chem.* **2019**.

(4) Gruzman, D.; Karton, A.; Martin, J. M. L. Performance of Ab Initio and Density Functional Methods for Conformational Equilibria of C n H 2 n +2 Alkane Isomers (n = 4−8) †. *J. Phys. Chem. A* **2009**, *113*, 11974–11983.

(5) Grimme, S.; Antony, J.; Ehrlich, S.; Krieg, H. A Consistent and Accurate Ab Initio Parametrization of Density Functional Dispersion Correction (DFT-D) for the 94 Elements H-Pu. *J. Chem. Phys.* **2010**, *132*, 154104.

(6) Lao, K. U.; Schäffer, R.; Jansen, G.; Herbert, J. M. Accurate Description of Intermolecular Interactions Involving Ions Using Symmetry-Adapted Perturbation Theory. *J. Chem. Theory Comput.* **2015**, *11*, 2473–2486.

(7) Yu, H.; Truhlar, D. G. Components of the Bond Energy in Polar Diatomic Molecules, Radicals, and Ions Formed by Group-1 and Group-2 Metal Atoms. *J. Chem. Theory Comput.* **2015**, *11*, 2968–2983.

(8) Kesharwani, M. K.; Karton, A.; Martin, J. M. L. Benchmark Ab Initio Conformational Energies for the Proteinogenic Amino Acids through Explicitly Correlated Methods. Assessment of Density Functional Methods. *J. Chem. Theory Comput.* **2016**, *12*, 444–454.

(9) Goerigk, L.; Grimme, S. A General Database for Main Group Thermochemistry, Kinetics, and Noncovalent Interactions − Assessment of Common and Reparameterized (Meta -)GGA Density Functionals. *J. Chem. Theory Comput.* **2010**, *6*, 107–126.

(10) Zhao, Y.; Lynch, B. J.; Truhlar, D. G. Multi-Coefficient Extrapolated Density Functional Theory for Thermochemistry and Thermochemical Kinetics. *Phys. Chem. Chem. Phys.* **2005**, *7*, 43.

(11) Zhao, Y.; González-Garda, N.; Truhlar, D. G. Benchmark Database of Barrier Heights for

Heavy Atom Transfer, Nucleophilic Substitution, Association, and Unimolecular Reactions and Its Use to Test Theoretical Methods. *J. Phys. Chem. A* **2005**, *109*, 2012–2018.
(12) Goerigk, L.; Grimme, S.; Chemie, T. O. A General Database for Main Group Thermochemistry , Kinetics , and Non-Covalent Interactions – Assessment of Common and Reparameterized (Meta-) GGA Density Functionals Supporting Information. **2009**, 1–32.
(13) Guner, V.; Khuong, K. S.; Leach, A. G.; Lee, P. S.; Bartberger, M. D.; Houk, K. N. A Standard Set of Pericyclic Reactions of Hydrocarbons for the Benchmarking of Computational Methods : The Performance of Ab Initio , Density Functional , CASSCF , CASPT2 , and CBS-QB3 Methods for the Prediction of Activation Barriers , Reaction Energetic. **2003**, 11445–11459.
(14) Ess, D. H.; Houk, K. N. Activation Energies of Pericyclic Reactions : Performance of DFT , MP2 , and CBS-QB3 Methods for the Prediction of Activation Barriers and Reaction Energetics of 1 , 3-Dipolar Cycloadditions , and Revised Activation Enthalpies for a Standard Set of Hydroc. **2005**, 9542–9553.
(15) Dinadayalane, T. C.; Vijaya, R.; Smitha, A.; Sastry, G. N. Diels−Alder Reactivity of Butadiene and Cyclic Five-Membered Dienes ((CH) 4 X, X = CH 2 , SiH 2 , O, NH, PH, and S) with Ethylene: A Benchmark Study. *J. Phys. Chem. A* **2002**, *106*, 1627–1633.
(16) Steinmann, S. N.; Csonka, G.; Corminboeuf, C. Unified Inter- and Intramolecular Dispersion Correction Functional Theory. *J. Chem. Theory Comput.* **2009**, *5*, 2950–2958.
(17) Krieg, H.; Grimme, S. Thermochemical Benchmarking of Hydrocarbon Bond Separation Reaction Energies: Jacob's Ladder Is Not Reversed! *Mol. Phys.* **2010**, *108*, 2655–2666.
(18) Kozuch, S.; Bachrach, S. M.; Martin, J. M. L. Conformational Equilibria in Butane-1,4-Diol: A Benchmark of a Prototypical System with Strong Intramolecular H - Bonds. **2014**.
(19) Sure, R.; Hansen, A.; Schwerdtfeger, P.; Grimme, S. Comprehensive Theoretical Study of All 1812 C60isomers. *Phys. Chem. Chem. Phys.* **2017**, *19*, 14296–14305.
(20) Yu, L.; Karton, A. Assessment of Theoretical Procedures for a Diverse Set of Isomerization Reactions Involving Double-Bond Migration in Conjugated Dienes. *Chem. Phys.* **2014**, *441*, 166–177.
(21) Johnson, E. R.; Mori-Sánchez, P.; Cohen, A. J.; Yang, W. Delocalization Errors in Density Functionals and Implications for Main-Group Thermochemistry. *J. Chem. Phys.* **2008**, *129*, 204112.
(22) Zhao, Y.; Truhlar, D. G. The M06 Suite of Density Functionals for Main Group Thermochemistry, Thermochemical Kinetics, Noncovalent Interactions, Excited States, and Transition Elements: Two New Functionals and Systematic Testing of Four M06-Class Functionals and 12 Other Function. *Theor. Chem. Acc.* **2008**, *120*, 215–241.
(23) Grimme, S. Semiempirical Hybrid Density Functional with Perturbative Second-Order Correlation. *J. Chem. Phys.* **2006**, *124*, 0–16.
(24) Grimme, S.; Mück-Lichtenfeld, C.; Würthwein, E. U.; Ehlers, A. W.; Goumans, T. P. M.; Lammertsma, K. Consistent Theoretical Description of 1,3-Dipolar Cycloaddition Reactions. *J. Phys. Chem. A* **2006**, *110*, 2583–2586.
(25) Piacenza, M.; Grimme, S. Systematic Quantum Chemical Study of DNA-Base Tautomers. *J. Comput. Chem.* **2004**, *25*, 83–98.
(26) Woodcock, H. L.; Schaefer, H. F.; Schreiner, P. R. Problematic Energy Differences between

Cumulenes and Poly-Ynes: Does This Point to a Systematic Improvement of Density Functional Theory? *J. Phys. Chem. A* **2002**, *106*, 11923–11931.
(27) Schreiner, P. R.; Fokin, A. A.; Pascal, R. A.; Meijere, A. De. Many Density Functional Theory Approaches Fail To Give Reliable Large Hydrocarbon Isomer Energy Differences. **2006**, 10–13.
(28) Lepetit, C.; Chermette, H.; Heully, J.; Lyon, D.; Uni, V.; Umr, C. Description of Carbo - Oxocarbons and Assessment of Exchange-Correlation Functionals for the DFT Description of Carbo -Mers. **2007**, 136–149.
(29) Lee, J. S. Accurate Ab Initio Binding Energies of Alkaline Earth Metal Clusters. *J. Phys. Chem. A* **2005**, *109*, 11927–11932.
(30) Karton, A.; Martin, J. M. L. Explicitly Correlated Benchmark Calculations on C 8 H 8 Isomer Energy Separations : How Accurate Are DFT , Double-Hybrid , and Composite Ab Initio Procedures ? **2012**, *8976*.
(31) Zhao, Y.; Tishchenko, O.; Gour, J. R.; Li, W.; Lutz, J. J.; Piecuch, P.; Truhlar, D. G. Thermochemical Kinetics for Multireference Systems: Addition Reactions of Ozone. *J. Phys. Chem. A* **2009**, *113*, 5786–5799.
(32) Manna, D.; Martin, J. M. L. What Are the Ground State Structures of C 20 and C 24 ? An Explicitly Correlated Ab Initio Approach. **2016**.
(33) Friedrich, J.; Hänchen, J. Incremental CCSD(T)(F12*)|MP2: A Black Box Method to Obtain Highly Accurate Reaction Energies. *J. Chem. Theory Comput.* **2013**, *9*, 5381–5394.
(34) Friedrich, J. Efficient Calculation of Accurate Reaction Energies—Assessment of Different Models in Electronic Structure Theory. *J. Chem. Theory Comput.* **2015**, *11*, 3596–3609.
(35) You, A.; Be, M. A. Y.; In, I. Gaussian-2 Theory for Molecular Energies of First- and Second-Row Compounds Compounds. **1998**, *7221*.
(36) Curtiss, L. A.; Redfern, P. C. Assessment of Gaussian-2 and Density Functional Theories for the Computation of Enthalpies of Formation for the Computation of Enthalpies of Formation. **2000**, *1063*.
(37) Kozuch, S.; Martin, J. M. L. Halogen Bonds: Benchmarks and Theoretical Analysis. *J. Chem. Theory Comput.* **2013**, *9*, 1918–1931.
(38) Řezáč, J.; Riley, K. E.; Hobza, P. Benchmark Calculations of Noncovalent Interactions of Halogenated Molecules. *J. Chem. Theory Comput.* **2012**, *8*, 4285–4292.
(39) Schwabe, T.; Grimme, S. Double-Hybrid Density Functionals with Long-Range Dispersion Corrections: Higher Accuracy and Extended Applicability. *Phys. Chem. Chem. Phys.* **2007**, *9*, 3397–3406.
(40) Grimme, S. Seemingly Simple Stereoelectronic Effects in Alkane Isomers and the Implications for Kohn–Sham Density Functional Theory. *Angew. Chemie Int. Ed.* **2006**, *45*, 4460–4464.
(41) Goerigk, L.; Grimme, S. Efficient and Accurate Double-Hybrid-Meta-GGA Density Functionals—Evaluation with the Extended GMTKN30 Database for General Main Group Thermochemistry, Kinetics, and Noncovalent Interactions. *J. Chem. Theory Comput.* **2011**, *7*, 291–309.
(42) Grimme, S.; Steinmetz, M.; Korth, M. How to Compute Isomerization Energies of Organic Molecules with Quantum Chemical Methods. *J. Org. Chem.* **2007**, *72*, 2118–2126.
(43) Goerigk, L.; Sharma, R. The INV24 Test Set: How Well Do Quantum-Chemical Methods

Describe Inversion and Racemization Barriers? *Can. J. Chem.* **2016**, *94*, 1133–1143.
(44) Huenerbein, R.; Schirmer, B.; Moellmann, J.; Grimme, S. Effects of London Dispersion on the Isomerization Reactions of Large Organic Molecules: A Density Functional Benchmark Study. *Phys. Chem. Chem. Phys.* **2010**, *12*, 6940–6948.
(45) Fogueri, U. R.; Kozuch, S.; Karton, A.; Martin, J. M. L. The Melatonin Conformer Space: Benchmark and Assessment of Wave Function and DFT Methods for a Paradigmatic Biological and Pharmacological Molecule. *J. Phys. Chem. A* **2013**, *117*, 2269–2277.
(46) Grimme, S.; Kruse, H.; Goerigk, L.; Erker, G. The Mechanism of Dihydrogen Activation by Frustrated Lewis Pairs Revisited. *Angew. Chemie Int. Ed.* **2010**, *49*, 1402–1405.
(47) Setiawan, D.; Kraka, E.; Cremer, D. Strength of the Pnicogen Bond in Complexes Involving Group Va Elements N, P, and As. **2015**.
(48) Karton, A.; O'Reilly, R. J.; Chan, B.; Radom, L. Determination of Barrier Heights for Proton Exchange in Small Water, Ammonia, and Hydrogen Fluoride Clusters with G4(MP2)-Type, MPn, and SCS-MPn Procedures-a Caveat. *J. Chem. Theory Comput.* **2012**, *8*, 3128–3136.
(49) Neese, F.; Schwabe, T.; Kossmann, S.; Schirmer, B.; Grimme, S. Assessment of Orbital-Optimized , Spin-Component Scaled Second-Order Many-Body Perturbation Theory for Thermochemistry and Kinetics. **2009**, 3060–3073.
(50) Jurečka, P.; Šponer, J.; Černý, J.; Hobza, P. Benchmark Database of Accurate (MP2 and CCSD(T) Complete Basis Set Limit) Interaction Energies of Small Model Complexes, DNA Base Pairs, and Amino Acid Pairs. *Phys. Chem. Chem. Phys.* **2006**, *8*, 1985–1993.
(51) Řezáč, J.; Riley, K. E.; Hobza, P. S66: A Well-Balanced Database of Benchmark Interaction Energies Relevant to Biomolecular Structures. *J. Chem. Theory Comput.* **2011**, *7*, 2427–2438.
(52) French, A. D.; Johnson, G. P.; Stortz, C. A. Evaluation of Density Functionals and Basis Sets for Ga. **2009**, 679–692.
(53) Karton, A.; Rabinovich, E.; Martin, J. M. L.; Ruscic, B. W4 Theory for Computational Thermochemistry: In Pursuit of Confident Sub-KJ/Mol Predictions. *J. Chem. Phys.* **2006**, *125*, 144108.
(54) Kruse, H.; Mladek, A.; Gkionis, K.; Hansen, A.; Grimme, S.; Sponer, J. Quantum Chemical Benchmark Study on 46 RNA Backbone Families Using a Dinucleotide Unit. *J. Chem. Theory Comput.* **2015**, *11*, 4972–4991.
(55) Karton, A.; Daon, S.; Martin, J. M. L. W4-11: A High-Confidence Benchmark Dataset for Computational Thermochemistry Derived from First-Principles W4 Data. *Chem. Phys. Lett.* **2011**, *510*, 165–178.
(56) Iii, W. A. G.; Park, U. V; Pennsyl, V. Evaluation of B3LYP , X3LYP , and M06-Class Density Functionals for Predicting the Binding Energies of Neutral , Protonated , and Deprotonated Water Clusters. **2009**, 1016–1026.
(57) Karton, A.; O'Reilly, R. J.; Radom, L. Assessment of Theoretical Procedures for Calculating Barrier Heights for a Diverse Set of Water-Catalyzed Proton-Transfer Reactions. *J. Phys. Chem. A* **2012**, *116*, 4211–4221.
(58) Zhao, Y.; Ng, H. T.; Peverati, R.; Truhlar, D. G. Benchmark Database for Ylidic Bond Dissociation Energies and Its Use for Assessments of Electronic Structure Methods. *J. Chem. Theory Comput.* **2012**, *8*, 2824–2834.
(59) Cao, J.; Berne, B. J. Many-body Dispersion Forces of Polarizable Clusters and Liquids. *J.*

Chem. Phys. **1992**, *97*, 8628–8636.
(60) Hermann, J.; DiStasio, R. A.; Tkatchenko, A. First-Principles Models for van Der Waals Interactions in Molecules and Materials: Concepts, Theory, and Applications. *Chem. Rev.* **2017**, *117*, 4714–4758.
(61) Santra, G.; Sylvetsky, N.; Martin, J. M. L. Minimally Empirical Double-Hybrid Functionals Trained against the GMTKN55 Database: RevDSD-PBEP86-D4, RevDOD-PBE-D4, and DOD-SCAN-D4. *J. Phys. Chem. A* **2019**, *123*, 5129–5143.
(62) Karton, A.; Martin, J. M. L. Explicitly Correlated Wn Theory: W1-F12 and W2-F12. *J. Chem. Phys.* **2012**, *136*, 124114.
(63) Weigend, F.; Ahlrichs, R. Balanced Basis Sets of Split Valence, Triple Zeta Valence and Quadruple Zeta Valence Quality for H to Rn: Design and Assessment of Accuracy. *Phys. Chem. Chem. Phys.* **2005**, *7*, 3297–3305.
(64) Sancho-García, J. C.; Brémond; Savarese, M.; Pérez-Jiménez, A. J.; Adamo, C. Partnering Dispersion Corrections with Modern Parameter-Free Double-Hybrid Density Functionals. *Phys. Chem. Chem. Phys.* **2017**, *19*, 13481–13487.
(65) Hui, K.; Chai, J. Da. SCAN-Based Hybrid and Double-Hybrid Density Functionals from Models without Fitted Parameters. *J. Chem. Phys.* **2016**, *144*, 044114.
(66) Yanai, T.; Tew, D. P.; Handy, N. C. A New Hybrid Exchange–Correlation Functional Using the Coulomb-Attenuating Method (CAM-B3LYP). *Chem. Phys. Lett.* **2004**, *393*, 51–57.
(67) Verma, P.; Wang, Y.; Ghosh, S.; He, X.; Truhlar, D. G. Revised M11 Exchange-Correlation Functional for Electronic Excitation Energies and Ground-State Properties. *J. Phys. Chem. A* **2019**, *123*, 2966–2990.
(68) Santra, G.; Martin, J. M. L. Some Observations on the Performance of the Most Recent Exchange-Correlation Functionals for the Large and Chemically Diverse GMTKN55 Benchmark. In *AIP Conference Proceedings*; 2019; p 030004.
(69) Adamo, C.; Barone, V. Toward Reliable Density Functional Methods without Adjustable Parameters: The PBE0 Model. *J. Chem. Phys.* **1999**, *110*, 6158–6170.
(70) Zhao, Y.; Truhlar, D. G. Design of Density Functionals That Are Broadly Accurate for Thermochemistry, Thermochemical Kinetics, and Nonbonded Interactions. *J. Phys. Chem. A* **2005**, *109*, 5656–5667.
(71) Becke, A. D. A New Mixing of Hartree-Fock and Local Density-Functional Theories. *J. Chem. Phys.* **1993**, *98*, 1372–1377.
(72) Mardirossian, N.; Head-Gordon, M. Mapping the Genome of Meta-Generalized Gradient Approximation Density Functionals: The Search for B97M-V. *J. Chem. Phys.* **2015**, *142*, 074111.
(73) Sun, J.; Ruzsinszky, A.; Perdew, J. Strongly Constrained and Appropriately Normed Semilocal Density Functional. *Phys. Rev. Lett.* **2015**, *115*, 036402.
(74) Perdew, J. P.; Ruzsinszky, A.; Csonka, G. I.; Constantin, L. A.; Sun, J. Workhorse Semilocal Density Functional for Condensed Matter Physics and Quantum Chemistry. *Phys. Rev. Lett.* **2009**, *103*, 026403.
(75) Perdew, J. P.; Burke, K.; Ernzerhof, M. Generalized Gradient Approximation Made Simple. *Phys. Rev. Lett.* **1996**, *77*, 3865–3868.
(76) Perdew, J. P.; Ernzerhof, M.; Burke, K. [ERRATA] Generalized Gradient Approximation Made Simple. *Phys. Rev. Lett.* **1996**, *77*, 3865–3868.

(77) Slater, J. C. A Simplification of the Hartree-Fock Method. *Phys. Rev.* **1951**, *81*, 385–390.
(78) Perdew, J. P.; Wang, Y. Accurate and Simple Analytic Representation of the Electron-Gas Correlation Energy. *Phys. Rev. B* **1992**, *45*, 13244–13249.
(79) Martin, J. M. L.; Santra, G. Empirical Double-Hybrid Density Functional Theory: A 'Third Way' in Between WFT and DFT. *Isr. J. Chem.* **2020**, *60*, 787–804.
